# Ionotropic Receptor Genes in Fig Wasps: Evolutionary Insights from Comparative Studies

**DOI:** 10.3390/insects16070679

**Published:** 2025-06-29

**Authors:** Hui Yu, Xiaojue Nong, Weicheng Huang, Ling Yang, Chantarasuwan Bhanumas, Yongmei Xiong, Seping Dai

**Affiliations:** 1 Key Laboratory of National Forestry and Grassland Administration on Plant Conservation and Utilization in Southern China, South China Botanical Garden, Chinese Academy of Sciences, Guangzhou 510650, China; nongxiaojue@scbg.ac.cn (X.N.); huangwc0921@163.com (W.H.); yangling20030109@163.com (L.Y.); 2 Guangdong Provincial Key Laboratory of Applied Botany, South China Botanical Garden, Chinese Academy of Sciences, Guangzhou 510650, China; 3 Thailand Natural History Museum, National Science Museum, Ptthum Thani 12120, Thailand; b.chantarasuwan@gmail.com; 4 Guangzhou Institute of Forestry and Landscape Architecture, Guangzhou 510405, China; ymxiong1004@126.com (Y.X.); gzifla_dsp@gz.gov.cn (S.D.)

**Keywords:** ionotropic receptors, chemical communication, evolution, *Ficus*, fig wasps, mutualism

## Abstract

The mechanisms of chemoreception in fig wasps (Hymenoptera, Agaonidae) are of primary importance in their co-evolutionary relationship with the fig trees they pollinate. As the supplementary receptors to odorant receptors (ORs) and gustatory receptors (GRs) in insects, we compare the evolutionary characters of ionotropic receptors (IRs) among 25 fig wasp taxa in six genera. In total, we identified 205 IRs in 25 fig wasps, with each taxon recording from 5 to 12 IR genes. We found 189 IR genes clustered into 18 orthologous groups that can be divided into three types: IRco, antennal IRs, and divergent IRs. More IRs belong to antennal IRs in fig wasps, which can be sensitive to acids, aldehydes, polyamines, salt, amino acids, and temperature/humidity according to homology comparison. Additionally, some IR genes in fig wasps do not cluster with those of outgroup species (e.g., *Drosophila melanogaster*, *Apis mellifera*), suggesting they may represent a unique group and may have special functions in fig wasps. Divergent IRs are very few, with large sequence variation between species. Compared to ORs and GRs in fig wasps, gene sequences in most IR orthologous groups are more conserved between genera, with the lowest sequence similarity in 10 orthologous groups (including three IRco) exhibiting above 58.5%. Gene sequences are consistent with the phylogenetic relationships among fig wasps, which is the same as ORs and GRs. Strong purifying selection of IR genes was detected, as shown by the low ω values. Signatures of positive selection were detected in loci from three orthologous groups. Our results provide important molecular information for further studies on chemosensory mechanisms in fig wasps.

## Simple Summary

Fig wasps mainly depend on the specific chemical cues released by receptive syconia to find their fig hosts. However, we know little about the molecular mechanisms of chemosensation in fig wasps. This study compares the evolutionary characters of ionotropic receptors (IRs) among 25 fig wasp taxa with three types: IRco, antennal IRs, and divergent IRs. There are considerable differences in IR gene sequences between species, which are consistent with the phylogenetic relationships among fig wasps. In addition, strong purifying selection of IRs was found. The results give us a better understanding of the molecular basis of the peripheral chemosensory system in fig wasps.

## 1. Introduction

Fig trees (*Ficus*, Moraceae) and pollinating fig wasps (Agaonidae) constitute the most closely related mutually beneficial symbiosis known so far. Fig trees are distributed in tropical and subtropical forests, with complex ecological environments and rich biodiversity [1]. They rely on fig wasps for pollination, and also provide places for the reproduction and development of fig wasps [2]. Although there are about 750 species in *Ficus*, they all have an enclosed inflorescence (also known as a syconium) with one ostiole connecting it with the outside [1]. Attracted by the specific volatile compounds (VOCs) emitted by the ostiole of the host fig, adult female fig wasps enter receptive syconia, laying eggs while also pollinating the host. After a period of time, the egg develops into an adult, and after mating, the female fig wasp flies out of the primary syconium to find another receptive syconium to begin a new life cycle [2].

Olfaction plays an important role in the production and maintenance of the highly specialized symbiotic system of fig wasps. Fig wasps mainly rely on the accurate identification of specific VOCs released from receptive syconia of the obligate fig, and distinguish them from other odors in the environment to locate oviposition sites [3]. The syconium is equivalent to the ‘grave’ of the pollinator, exerting strong selective pressure on fig wasps to identify host VOCs and enter the same fig species as the original syconium [4]. Conversely, it also promotes the species specificity of VOCs in figs [5], ensuring the hosts emit only a few stable compounds during the receptive phase at specific times to simplify the signal, which facilitates identification by specific pollinators [3,6]. Female fig wasps possess antennae rich in olfactory receptors [7] and can effectively identify their host’s specific VOCs, showing a highly significant preference for selection [6].

Insect olfactory receptors mainly include two types: odor receptors (ORs) and ionotropic receptors (IRs) [8]. While many studies have focused on the functions of insect ORs [9,10,11], research on IRs has primarily centered on the model insect *Drosophila melanogaster* [8,12,13,14,15,16,17]. Studies on other insects are limited; for example, investigations into fig wasp IRs have mainly focused on gene identification [6,18,19,20,21]. The number of IRs in fig wasp genomes ranges from 11 to 30, similar to that of golden wasp *Nasonia vitripennis* and honey bee *Apis mellifera*, ranging from 9 to 29 [18,21], but lower than in the ant *Zootermopsis nevadensis*, mosquito *Aedes aegypti,* and *Drosophila melanogaster*, which contain 140, 95, and 66 IRs, respectively [8,14,22].

IRs have an extracellular N-terminus, a bipartite ligand-binding domain (with two lobes, S1 and S2, separated by an ion channel domain), and a short cytoplasmic C-terminus [23]. Given that amino acid sequence similarity within *D*. *melanogaster* IRs varies from 10% to 70%, it can be inferred that IRs have diverse functions in insects [8]. Based on sequence identity and gene expression, the 66 IRs in the *D. melanogaster* genome can be divided into three subfamilies: co-receptor IRs (IRcos), antennal IRs, and divergent IRs [8]. IRcos (including IR8a, IR25a, and IR76b) primarily function through co-expression with other IRs and are similar to ORcos [12,24]. Antennal IRs (also called olfactory IRs) are mostly specifically expressed in antennae and show low or no expression in other organs/tissues. *D. melanogaster* has 16 antennal IRs, which play important roles in sensing polyamines and acids [12,25,26] as well as temperature/humidity [16,27]. Additionally, antennal IRs may be related to insect foraging and courtship behavior [12]. Homologous genes of antennal IRs exist in many insects, suggesting these genes are relatively conserved across species. Divergent IRs are mainly expressed in non-antennal organs/tissues (e.g., taste receptor neurons), indicating their potential role in taste sensing [17]. These IRs are species-specific, with highly divergent amino acid sequences both within and between species.

Antennal IRs can detect acids, aldehydes, and amines in the environment, and play a complementary role with ORs in VOCs recognition [12]. ORs and antennal IRs are expressed in developmentally distinct sensory lineages in the antenna: ORs are mainly expressed in basiconic and trichoid sensilla, while antennal IRs are primarily expressed in the coeloconic sensilla of *D. melanogaster*, *Anopheles gambiae*, *Schistocerca gregaria*, and *Mythimna separata* [8,28,29,30]. The relatively strong ligands of IRs can only elicit weak responses of ORs or may not be recognized by them at all, whereas the strongest OR ligands (mainly esters, alcohols, and ketones) generally fail to activate IRs, with some exceptions [26,31,32].

We have identified ORs and GRs in fig wasps [33]. The amino acid sequences within each OR and GR group vary significantly between species but align with the phylogenetic relationships among fig wasps. Strong purifying selection was detected in ORs and GRs, yet positive selection was also detected at certain loci. This suggests that fig wasp ORs can rapidly evolve to adapt to ecological pressures and play a crucial role in host-specific adaptation. While IRs are important complements to olfaction and taste, their identification, molecular basis, and functional roles in fig wasps remain poorly understood.

In this study, IR genes were identified in the transcriptomes of 25 fig wasp taxa [19], and sequence alignment and phylogenetic analysis were performed to characterize these putative IRs. The results are discussed in terms of the role that IRs play as a supplement to ORs in odor detection and have some other important functions, such as taste and temperature/humidity sensing, which contribute to maintaining the mutually beneficial symbiotic relationship between figs and fig wasps.

## 2. Materials and Methods

### 2.1. Gene Identification

High-quality transcriptomes were successfully sequenced for 25 fig wasp taxa (all adult females) representing 6 genera according to 22 *Ficus* host species (Chen et al., 2021 [19]; Table 1). Unlike previous studies that relied solely on Hmmer (http://www.hmmer.org/) to identify IR genes [19], this study employed both Blastp and Hmmer (http://www.hmmer.org/) to search for candidate IR genes based on 25 transcriptomes. We conducted Blastp searches (E-value < 10^−5^) to identify predicted genes using *Drosophila melanogaster*, *Apis mellifera*, *Apis cerana*, and *Nasonia vitripennis* IRs as input sequences. Based on the structural characteristics of IRs (PF00060) from the Pfam database (https://pfam.xfam.org/), we searched for candidate IR genes in the 25 fig wasp transcriptomes using the hmmscan command in HMM v3.3.2 (E-value < 10^−5^ and 25% HMM coverage). Finally, only candidate genes with conserved domains were retained using InterProScan [34] and the NCBI CDD web server (http://www.ncbi.nlm.nih.gov/Structure/cdd/wrpsb.cgi; accessed on 9 November 2019).

### 2.2. Orthologous Analysis of the IRs

Protein sequences were aligned using Clustal X [35]. Orthologous groups were predicted by orthoMCL [36]. The similarity of amino acid sequences in each orthologous group was compared by MegAlign in DNAStar at four levels: (1) same species but on different hosts; (2) related species within one fig host; (3) congeneric species; and (4) between genera (Table 1; [34]).

### 2.3. Phylogenetic Analysis of the IRs

The phylogenetic tree was constructed with the protein sequences of the candidate fig wasp IRs together with IRs from other insects, including *Drosophila melanogaster* [8], *Apis mellifera*, *Apis cerana,* and *Nasonia vitripennis* [18,22]. The tree was built using MEGA7.0 with the maximum likelihood method and Poisson correction for distance [37]. Node support was assessed via bootstrap analysis with 1000 replicates.

### 2.4. Tests of Positive Selection

Two models from the CodeML program in the PAML package v4.6 [38] were used to test the selective pressures on IR genes. Analyses were based on the phylogenetic tree of 25 fig wasps constructed from 625 single-copy orthologous genes [19], with each orthologous gene compared across at least four species.

For the branch model analysis [38], we first assumed that each branch in the phylogenetic tree has its own independent ω value. Using a free-ratio model, we calculated the ω values for each branch as the H1 hypothesis. Next, we used a one-ratio model (H0 hypothesis) to calculate a single ω value across all branches, assuming equal selection pressure. Finally, we compared the significance of the likelihood values between the two hypotheses using a chi-square test. A significant difference indicates heterogeneous ω values and branch-specific selection pressures.

Selection can act on both specific branches and amino acid sites. Branch-site models simultaneously analyze ω values for branches and sites, enabling detection of positive selection at both levels. Based on the phylogenetic tree, we set the null hypothesis model A (model = 2, nssites = 2, fix_omega = 1, omega = 1) as no selection and model A (model = 2, nssites = 2, fix_omega = 0, omega = 2) as positive selection. Likelihood ratio tests (LRTs) compared hypotheses, with significance determined via chi-square tests. For significant LRT results, we applied the Bayes Empirical Bayes (BEB) analysis to identify positively selected sites with >95% confidence.

## 3. Results

### 3.1. Identification and Phylogenetic Analysis of IR Genes

Out of 25 fig wasps, we identified a total of 205 IRs, with a range of 4 to 12 (Mean 8.2 ± SE 1.73) IRs for each taxa (Table 1). The length of IRs ranged from 105aa to 931aa, with an average of 323aa (Table S1). The proportion of IRs with at least two transmembrane domains was 52.2%.

Four of the five *Blastophaga* taxa from different hosts collected in Guangdong province are currently considered to belong to a single species [39], while *Blastophaga* 4—reared from *F. pyriformis* in Thailand—may be a different, but related, species. *Valisia javana* species number designations correspond with those in [40]. *Ficus hirta* is host to at least nine *Valisia* species, eight of which share a recent common ancestor [40]. We selected four of them (*Valisia javana* complex sp. 1, sp. 2, sp. 7, and sp. 8) and look at them as related species in one host fig.

**Figure 1:**
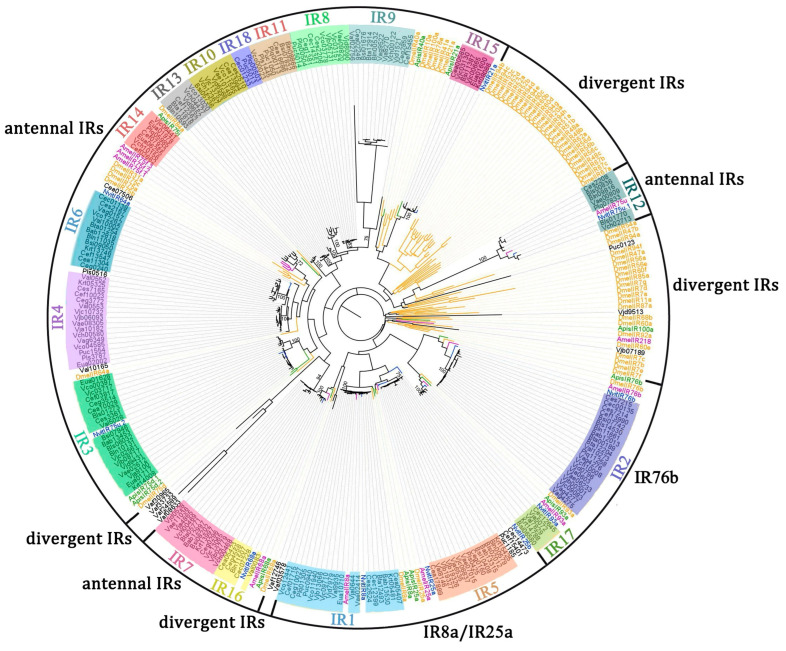
Phylogenetic tree of IR genes from fig wasps, *Drosophila melanogaster*, *Nasonia vitripennis*, *Apis cerana,* and *Apis mellifera*. Clades with black color indicate IRs of fig wasps. Clades with a yellow color indicate IR genes of *Drosophila melanogaster*, those with a blue color indicate IR genes of *Nasonia vitripennis*, those with a green color indicate IR genes of *Apis cerana*, and those with a purple color indicate IR genes of *Apis mellifera*. These IRs can be divided into three types: IRco (IR8a and IR25a), antennal IRs, and divergent IRs. IR1–18 with different colors represent the orthologous groups of fig wasps.

The phylogenetic analyses were constructed based on IRs of the fig wasps and those of *D. melanogaster*, *A. mellifera*, *A. cerana*, and *N. vitripennis* as outgroups. In the phylogenetic tree, IR1, IR2, and IR5 clustered into the branches of IR8a, IR76b, and IR25a, respectively (Figure 1), which are the Ircos of *D. melanogaster*, *A. mellifera*, *A. cerana,* and *N. vitripennis*. The remaining Irs genes of fig wasps can be clustered into antennal Irs and divergent Irs according to the IRs of *D. melanogaster*, *A. mellifera*, *A. cerana*, and *N. vitripennis* (Figure 1).

IR3, IR12, and IR14–17 converge in the same branch as the IRs of four outgroup species whose functions have been predicted previously (Figure 1). However, IR7–10, IR11, IR13, and IR18 cannot be clustered in the same branch as IRs from the outgroups, and should be a unique group in fig wasps.

### 3.2. Analysis of Orthologous IR Genes

For the 205 IRs, 189 were clustered into 18 orthologous groups using orthoMCL (Table 1; Figure 1). No orthologous group was recorded in all species (Table 1), but IR1 was present in 18 of the 25 fig wasps, and IR2–5 were present in more than half of the species. IR1, IR2, and IR5 are IRco and conserved between species in orthologous groups with sequence similarities of 70.9–100%, 63.5–100%, and 67.9–100% (Table 2). For the other orthologous groups, the genes in IR4, IR6–10, and IR16 are conserved, with sequence similarity between genera exhibiting above 58.5%, while those in the other groups are as low as 11.7% to 45.3% (Table 2).

**Figure 2:**
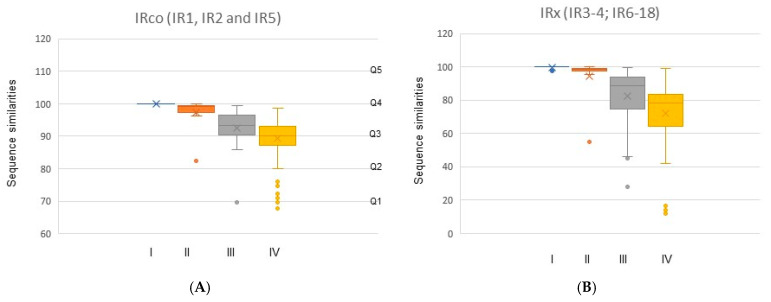
Sequence similarities of IRs in 25 fig wasp taxa associated with four levels of taxonomic proximity. I (blue) represent genes between same species but on four different hosts; II (red) represent genes between related species across different sites; III (grey) represent genes between species within one genus but except related species; IV (yellow) represent genes between genera. Q1 represents the lower whisk limit and is the minimum data point extending to 1.5 times the frame height from the bottom of the frame; Q2 indicates that 25% of the data is less than this value; Q3 is the median data; Q4 indicates that 75% of the data is less than or equal to this value; and Q5 represents the upper whisk limit and is the maximum data point extending to 1.5 times the frame height from the top of the frame. Values exceeding the upper or lower limit of the whisker will be represented by a dot: “·”.

Sequence similarities of both IRco and IRx between species are consistent with their phylogenetic relationship, which can be divided into four levels of taxonomic proximity (Table 2; Figure 2). In total, sequence similarity between species from highest to lowest is same species on different hosts, closely related species on the same host, congeneric species, and between genera. For *Blastophaga* taxa, sequence similarities of the same species on different hosts in each orthologous group are all 100% with the exception of IR9 (Table 2). Between the related species, the mean sequence similarity of IRco is 97.2 ± SE 4.95 (Figure 2A) and similar to those of IRx 94.2 ± 12.9 (Figure 2B). While for congeneric species and between genera, mean sequence similarities of IRco are 92.5 ± 5.90 and 85.5 ± 7.9, those of IRx are 82.2 ± 16.9 and 71.8 ± 16.3.

### 3.3. Selective Pressures on IR Genes

The genes in 17 IRs (IR1 to IR17) had the ratio of ω calculated. In general, the estimated ω values from a one-ratio model (assuming the same selective pressures on all amino acid sites) were low in all clades, ranging from 0.019 to 0.168, suggesting the existence of strong purifying selection (Table 3). Further comparison of ω among branches under free-ratio and one-ratio models yielded significant likelihood ratio tests for IR3, IR6, IR8, IR10, IR12, and IR15 (*p* < 0.05), indicating that there was substantial variation in evolutionary rates of the genes within these groups.

In the branch-site model, the BEB posterior probability of amino acid sites in some branches was greater than 95% in IR4, IR9, IR11, and IR15 (Table 4). Further analysis of model A versus null model A again detected sites where there was evidence of positive selection (*p* < 0.05) in them, except IR15 (Table 4).

## 4. Discussion

A total of 205 IRs were identified for 25 fig wasp taxa, with each taxa having 4–12 IRs, which is lower than the genomic identification of fig wasps with more than 20 IR genes [18,20,21,41]. This discrepancy aligns with similar observations for ORs and GRs in fig wasps, where transcriptomic surveys (using whole-body samples) generally detect fewer genes than genomic analysis [33,42]. The lower number of IR genes may additionally reflect technical limitations, as whole-body sampling could miss low-abundance transcripts that fall below detection thresholds.

The identified IRs of fig wasps can be divided into 18 orthologous groups. The sequence similarities of IRs between fig wasp species are consistent with their phylogenetic and taxonomic proximity, which is in accord with related pollinators more likely shared between related figs and the same section/subsection of figs usually pollinated by one genus of fig wasps [43,44]. Like ORs, IRs can be divided into IRco and IRx, but their sequences are more conserved than those of ORs [33]. For traditional IR genes, sequence similarities between the genera of half of the orthologous groups exceed 50%. This shows that they may have important functions in fig wasps, the IR neuron response spectrum may be narrow, and the types of sensed chemicals are few [26,45,46]. Meanwhile, for the other IR groups, the lowest sequence similarities between species are from 11.7% to 45.3% and show the diversity of these genes.

IRs in insects form ion channels by combining traditional receptors and co-receptors, enabling odor molecule identification and signal transduction [47]. Therefore, co-receptors should be highly conserved across different insects. Unlike the single olfactory co-receptor, IRs can utilize multiple co-receptors (such as IR8a, IR76b, and IR25a), which are selectively co-expressed with one or several other different IRs and act on insect sensory channels [8,12]. The IR8a/IR76b/IR25a is highly conserved and often clustered with the ionotropic glutamate receptor (iGluR) as the ancestral sequence of the IR family [22,24,26,48]. Phylogenetic analysis of 205 fig wasp IRs revealed that IR1, IR2, and IR5 exhibit high sequence similarity among fig wasp species and cluster with *D. melanogaster*, *A. mellifera*, *A. cerana,* and *N. vitripennis* IR8a, IR76b, and IR25a proteins. Thus, IR1, IR2, and IR5 likely function as IRco in fig wasps, collaborating with other IRx to mediate sensory processes.

In contrast to *D. melanogaster*, where most of the IRs (48 IRs) belong to divergent IRs [8], the majority of fig wasp IRs are antennal IRs. Ten orthologous groups of antennal IRs in fig wasps cluster with those of *D. melanogaster*, *A. mellifera*, *A. cerana,* and *N. vitripennis*, suggesting these genes are conserved and primarily involved in olfaction. Similar to Hymenoptera species (*A. mellifera*, *A. cerana,* and *N. vitripennis*), only a few fig wasp IRs are divergent IRs. Among 25 fig wasp species, *Valisia* cf. *filippina*, *Platyscapa* sp., *V. javana* sp. 2, and *V. javana* sp. 8 possess limited divergent IRs, potentially participating in taste perception [17]. Unlike antennal IRs, divergent IRs show no obvious inter-species homology and are highly dispersed in the phylogenetic tree, exhibiting extensive sequence variation within and between species, which contributes to their high species specificity.

Based on functional predictions, fig wasp antennal IRs can be categorized into four types: olfactory, taste, temperature/humidity, and unknown specific functions. *D. melanogaster* IR75a, b, c, d, 64a, and 84a detect environmental acids/aldehydes, crucial for selecting feeding/spawning sites [12,26,47]. Fig wasp IR3, IR4, IR6, and IR14 cluster with them, suggesting similar functions. In *D. melanogaster*, IR41a and IR76b co-expression detects polyamine-rich food, enhancing fertility by increasing egg production/hatching rates [25]. Fig wasp IR15 and IR2 cluster with DmelIR41a and DmelIR76b, respectively, implying their potential role in polyamine detection.

Beyond antennal expression, DmelIR76b is also found in the proboscis [8], where it acts as a Na^+^ channel to directly sense environmental salts, serving as a key taste receptor gene [49]. It is also broadly expressed in larval taste neurons and co-expressed with other IRs for amino acid sensing [17,22,24]. Thus, fig wasp IR2 could have such taste functions. The tissue-specific expression patterns of IR genes hint at their functional roles, necessitating validation through reverse transcription PCR (RT-PCR) or real-time quantitative PCR (qPCR) across different fig wasp tissues (especially antennae).

Fig wasp IR15, IR16, and IR17 cluster with IR21a, IR68a, and IR93a of *D. melanogaster*, *A. mellifera*, *A. cerana,* and *N. vitripennis*, respectively. IR21a is a key receptor for insects to avoid overheating due to the environment and protect themselves from damage. IR93a is an essential gene for *D. melanogaster* to sense external temperature and humidity. These two genes are co-expressed with IR25a to sense cold [27]. Environmental humidity affects the suitability and geographical distribution of insects. *D. melanogaster* humidity sensing requires the cooperation of three highly conserved IRs: IR25a, IR93a, and IR68a [50]. Therefore, fig wasps IR15, IR16, and IR17 may also have the function of sensing temperature and humidity.

The IR8/IR9/IR10/IR11/IR13/IR18 clades in fig wasps form distinct phylogenetic groups that do not cluster with those of *D. melanogaster*, *A. mellifera*, *A. cerana,* and *N. vitripennis*, suggesting these IRs may have evolved specialized functions in fig wasps. This lineage-specific divergence, coupled with the absence of homologous IRs in other insect species, highlights the role of gene duplication and neofunctionalization as a key mechanism driving evolutionary innovation in ancient insect lineages [51,52]. The IR gene family exhibits evidence of strong purifying selection, as indicated by relatively low ω values (0.019~0.168). Notably, the positive selection has been detected at fewer sites within IR4, IR9, and IR11 lineages. Comparative analysis with *D. melanogaster* suggests that IR4 may be involved in acid odor detection, whereas IR9 and IR11 might possess some unknown functions.

The ability of fig wasp IR genes to detect acids, aldehydes, and polyamines is linked to their role in recognizing host fig tree volatile organic compounds (VOCs). However, symbiotic relationships evolve under multiple factors (e.g., environmental changes and pollinator behavioral adaptations), meaning IR gene contributions likely interact with other genetic and environmental influences—making it challenging to isolate their specific effects in the current analysis.

This mechanism may also apply to other obligate symbiotic arthropods (e.g., parasitic wasps). While phylogenetic and sequence comparisons have inferred functions for some IR genes (e.g., IRco as a co-receptor and antennal IRs in olfaction), experimental validation remains limited. Notably, the functions of divergent IRs are still poorly understood.

## 5. Conclusions

In summary, to our knowledge, this study represents the first large-scale comparative analysis of IR genes across transcriptomes from 25 fig wasp taxa. Figs and fig wasps are used as a model system to study co-evolution and co-speciation for their highly species-specific mutualism. This specificity is mainly achieved through insects’ preference for specific volatiles released by the receptive figs of their usual hosts, as well as the other chemical sensations that contribute to their symbiosis. As a supplement to ORs and GRs, the diversity and evolution of IRs in fig wasps have been studied here. Gene sequences between species in each group are consistent with the phylogenetic relationships among fig wasps. IR genes can be divided into IRco, antennal IRs, and divergent IRs. Compared with similar genes of known function with those of outgroup species (e.g., *Drosophila melanogaster*, *Apis mellifera*), they can detect acids, aldehydes, polyamines, salt, amino acids, and external temperature and humidity. In general, IRs are under strong purifying selection. Positive selection was detected only on several loci. These findings provide molecular information for future investigations on the chemosensory mechanisms in fig wasps.

## Figures and Tables

**Table 1:** The number of ionotropic receptor genes in 18 orthologous gene groups (IR1–IR18) from 25 fig wasp taxa.

Fig Wasp Taxa	Host *Ficus*	Number of IRs in Each Orthologous Group (IR 1–18)																		T OG	T IR (1)	T IR (2)
		1	2	3	4	5	6	7	8	9	10	11	12	13	14	15	16	17	18			
*Blastophaga* 1	*F. abeli*	1	1	1	-	1	1	1	-	-	-	-	-	-	-	-	-	-	-	6	6	6
*Blastophaga* 2	*F. erecta* var. *beecheyana*	-	1	1	-	1	1	-	-	1	-	1	1	1	-	-	1	-	-	9	9	9
*Blastophaga* 3	*F. variolosa*	-	1	2	-	1	-	-	-	-	-	-	2	-	-	-	-	-	-	4	6	6
*Blastophaga* 4	*F. pyriformis*	-	1	1	-	1	1	1	-	1	1	1	-	-	-	1	-	1	-	10	10	10
*Blastophaga* 5	*F. formosa*	1	2	-	-	-	1	1	-	1	-	-	-	1	-	-	-	-	-	6	7	7
*Ceratosolen appendiculatus*	*F. variegata*	1	-	1	-	1	1	-	-	1	1	1	-	-	-	1	1	-	-	9	9	10 *
*C. constrictus*	*F. fistulosa*	1	1	-	-	-	1	-	1	-	-	-	-	-	-	-	1	1	1	7	7	8 *
*C. fusciceps*	*F. racemosa*	1	1	1	1	-	1	-	-	-	-	-	-	1	2	-	-	-	-	7	8	10 *
*C. gravelyi*	*F. semicordata*	1	-	1	1	1	1	1	1	1	-	-	-	-	-	-	1	-	-	9	9	9
*C. solmsi*	*F. hispida*	1	2	1	1	-	1	-	1	-	1	-	1	-	-	-	-	-	-	8	9	9
*Eupristina altissima*	*F. altissima*	1	-	2	1	-	-	-	-	-	-	-	-	-	2	1	-	-	-	5	7	7
*Kradibia tentacularis*	*F. montana*	1	-	1	1	1	1	1	-	-	-	-	-	-	-	1	-	1	-	8	8	8
*Platyscapa* sp. 1	*F. concinna*	1	-	-	1	-	-	-	1	-	-	1	-	-	-	-	-	-	1	5	5	7
*P. quadraticeps*	*F. religiosa*	1	1	-	-	-	-	-	1	-	-	1	1	-	1	-	-	-	1	7	7	7
*Platyscapa* sp. 2	*F. rumphii*	-	-	-	1	1	-	-	-	-	-	1	-	-	-	-	-	-	-	3	3	4 *
*Valisia esquirolianae*	*F. triloba*	1	1	1	1	1	-	1	1	-	1	-	1	-	-	-	-	-	-	9	9	9
*V.* cf. *filippina*	*F. ruficaulis* var. *antaoensis*	-	-	-	-	-	-	-	-	-	-	-	-	-	-	-	-	-	-	0	0	7 *
*V. malayana*	*F. grossularioides*	-	3	-	1	-	1	-	-	1	-	-	-	-	-	-	-	-	-	4	6	6
*Valisia* sp. 1	*F. langkokensis*	1	1	2	2	1	1	-	-	-	-	-	-	-	-	-	1	1	-	8	10	10
*V. medusa*	*F. chartacea*	-	1	-	1	-	-	2	-	1	1	-	1	1	1	-	-	-	-	8	9	9
*V. compacta*	*F. fulva*	2	-	1	1	-	1	-	1	-	1	1	-	1	-	-	-	-	-	8	9	9
*V. javana* sp. 1	*F. hirta*	1	1	1	1	1	-	1	-	1	-	-	-	-	-	-	-	1	-	8	8	8
*V. javana* sp. 2	*F. hirta*	1	1	1	1	2	-	1	1	-	-	-	-	-	-	-	-	-	-	7	8	9
*V. javana* sp. 7	*F. hirta*	1	1	2	1	1	-	1	1	-	1	-	-	-	1	2	-	-	-	10	12	12
*V. javana* sp. 8	*F. hirta*	1	1	1	-	-	-	1	1	1	1	-	-	1	-	-	-	-	-	8	8	9 *
Total number of IRs in each orthologous group		19	21	21	16	14	13	12	10	9	8	7	7	6	7	6	5	5	3			
Number of taxa where present		18	17	17	15	13	13	11	10	9	8	7	6	6	5	5	5	5	3		189	205
Mean																				6.9	7.6	8.2
SE																				2.4	2.4	1.7

T OG = the number of orthologous groups present. T IR (1) = the total number of IR genes in each species that could be clustered into orthologous groups. T IR (2) = the total number of IR genes including those that did not cluster in the orthologous groups (presence indicated by *).

**Table 2:** The sequence-based identities of ionotropic receptor genes in 18 orthologous gene groups (IR1–IR18) from 25 fig wasp taxa.

Sequence Identities of IRs (%)	Number of IRs in Each Orthologous Group (IR 1–18)																	
	1	2	3	4	5	6	7	8	9	10	11	12	13	14	15	16	17	18
In same *Blastophaga* sp.	100	100	100	-	100	100	100	-	97.4	-	-	100	100	-	-	-	-	-
In *Blastophaga* wasps	-	99.4	99.1–99.6	-	82.3–100	99.5–99.6	97.4–98.7	-	97.4–99.6	-	99.2	-	-	-	-	-	-	-
In *Ceratosolen* wasps	88.8–94	69.8–87.4	44.9–82.6	83.4–87	93.3	58.9–73.5	-	91.4–93.4	88.1	89	-	-	-	-	-	80.5–85.4	-	**-**
In *Platyscapa* wasps	96.4	-	-	88.6	-	-	-	92.6	-	-	54.3–69.5	-	-	-	-	-	-	94.1
In *V. javana* wasps	98.4–99.6	96.2–99.2	55–98.6	96–98.9	97.9–99.5	-	98–100	95.6–97	98.3	97.4	-	-	-	-	-	-	-	-
In *Valisia* wasps	91.2–96.7	85.8–98.7	49.7–99.1	80.9–99.1	92.5–99.3	79.1–95.6	91.7–99.4	89.9–97	86.7–95.3	93.3–98.7	-	47.1	28.8–85.9	54.2	-	69.6–85.4	85.2	-
Between genera in each group	70.9–98.7	63.5–83.8	41.7–90.2	72.6–89.7	67.9–96.8	59.1–81.3	68.1–86.6	75.4–92.6	64.4–89.2	83.1–88.1	41.7–99.2	44.5–77.1	12.8–85.8	45.3–81.7	-	69.6–84.6	11.7–85.2	14.5–14.9
In each group	70.9–100	63.5–100	41.7–100	72.6–99.1	67.9–100	58.5–100	68.1–100	75.4–97	64.4–99.6	83.1–98.7	41.7–99.2	44.5–100	12.8–100	45.3–81.7	20.7–72.3	69.6–85.4	11.7–85.2	14.5–94.1

**Table 3:** Nonsynonymous to synonymous substitution ratios (ω) of IRs in 25 fig wasp taxa.

Gene	Model	lnL H0 Versus H1	Mates of Parameter dN/dS (ω)	df	2ΔlnL	*p*-Value
IR1	Free-ratio	−1022.584	Variable ω	33	33.568	0.220
	One-ratio	−1039.368	ω = 0.035			
IR2	Free-ratio	−1244.001	Variable ω	31	27.050	0.335
	One-ratio	−1257.526	ω = 0.136			
IR3	Free-ratio	−2814.628	Variable ω	31	57.032	0.002 *
	One-ratio	−2843.144	ω = 0.057			
IR4	Free-ratio	−978.290	Variable ω	27	26.566	0.244
	One-ratio	−991.573	ω = 0.045			
IR5	Free-ratio	−726.358	Variable ω	23	12.932	0.477
	One-ratio	−732.824	ω = 0.022			
IR6	Free-ratio	−1934.165	Variable ω	23	42.834	0.004 *
	One-ratio	−1955.582	ω = 0.140			
IR7	Free-ratio	−2010.821	Variable ω	19	16.640	0.307
	One-ratio	−2019.141	ω = 0.079			
IR8	Free-ratio	−2159.311	Variable ω	17	34.518	0.004 *
	One-ratio	−2176.570	ω = 0.106			
IR9	Free-ratio	−792.710	Variable ω	15	8.348	0.455
	One-ratio	−796.884	ω = 0.059			
IR10	Free-ratio	−1823.814	Variable ω	13	33.150	0.001 *
	One-ratio	−1840.389	ω = 0.065			
IR11	Free-ratio	−185.188	Variable ω	11	13.518	0.130
	One-ratio	−191.947	ω = 0.168			
IR12	Free-ratio	−1686.123	Variable ω	9	22.448	0.004 *
	One-ratio	−1697.347	ω = 0.113			
IR13	Free-ratio	−105.870	Variable ω	9	2.156	0.494
	One-ratio	−106.948	ω = 0.019			
IR14	Free-ratio	−738.105	Variable ω	7	3.584	0.413
	One-ratio	739.897	ω = 0.109			
IR15	Free-ratio	−239.273	Variable ω	7	12.978	0.036 *
	One-ratio	−245.762	ω = 0.060			
IR16	Free-ratio	−737.614	Variable ω	7	9.294	0.116
	One-ratio	−742.261	ω = 0.120			
IR17	Free-ratio	−247.287	Variable ω	7	6.666	0.232
	One-ratio	−250.620	ω = 0.049			

* Significant within the 5% interval; 2ΔlnL: Likelihood ratio test.

**Table 4:** Positively selected sites on the IRs in 25 fig wasp taxa.

Gene	Clade	Model	lnL	Estimates of Parameters *d_N_*/*d_S_*	df	2ΔlnL	*p*-Value	Positively Selected Sites
IR4	2	Model A	−9787.966	ω0 = 0.065, ω1 = 1, ω2 = 999	1	61.022	0.000	46A *
		Null A	−9818.477	ω0 = 0.064, ω1 = 1, ω2 = 1				
	Vjb	Model A	−9815.376	ω0 = 0.063, ω1 = 1, ω2 = 1	1	0	0.500	317I *
		Null A	−9815.376	ω0 = 0.063, ω1 = 1, ω2 = 1				
IR9	Bta	Model A	−4218.911	ω0 = 0.070, ω1 = 1, ω2 = 999	1	30.338	0.000	51I **
		Null A	−4234.08	ω0 = 0.070, ω1 = 1, ω2 = 1				
IR11	Plq	Model A	−6009.32	ω0 = 0.129, ω1 = 1, ω2 = 4.622	1	6.672	0.005	330V *
		Null A	−6012.656	ω0 = 0.125, ω1 = 1, ω2 = 1				
IR15	Vjc	Model A	−3440.001	ω0 = 0.056, ω1 = 1, ω2 = 1.973	1	2.126	0.072	245F *, 249T *, 251T *, 310E **, 314Y **, 315R **, 361S **, 363A **
		Null A	−3441.06421	ω0 = 0.055, ω1 = 1, ω2 = 1				
	Krt	Model A	−3454.803	ω0 = 0.064, ω1 = 1, ω2 = 1	1	0	0.500	366W *

* Significant within the 5% interval; ** Significant within the 1% interval; 2ΔlnL: Likelihood ratio test.

## Data Availability

The original contributions presented in this study are included in the article/supplementary material. Further inquiries can be directed to the corresponding author.

## Supplementary Materials

The following supporting information can be downloaded at: https://www.mdpi.com/article/10.3390/insects16070679/s1, Table S1: The 205 IRs in 25 fig wasp taxa with numbers of amino acids and transmembrane domain, as well as their expression level (Fragments Per Kilobase of transcript per Million mapped reads; FPKM).

## References

[1] Berg C.C. (2003). Flora Malesiana Precursor for the Treatment of Moraceae 1: The Main Subdivision of Ficus: The Subgenera. Blumea.

[2] Galil J., Eisikowitch D. (1968). Flowering Cycles and Fruit Types of Ficus Sycomorus in Israel. New Phytol..

[3] Hossaert-McKey M., Soler C., Schatz B., Proffit M. (2010). Floral Scents: Their Roles in Nursery Pollination Mutualisms. Chemoecology.

[4] Moe A.M., Weiblen G.D. (2012). Pollinator-Mediated Reproductive Isolation among Dioecious Fig Species (Ficus, Moraceae). Evolution.

[5] Conchou L., Cabioch L., Rodriguez L.J., Kjellberg F. (2014). Daily Rhythm of Mutualistic Pollinator Activity and Scent Emission in Ficus Septica: Ecological Differentiation between Co-Occurring Pollinators and Potential Consequences for Chemical Communication and Facilitation of Host Speciation. PLoS ONE.

[6] Wang G., Zhang X., Herre E.A., McKey D., Machado C.A., Yu W.B., Cannon C.H., Arnold M.L., Pereira R.A.S., Ming R. (2021). Genomic Evidence of Prevalent Hybridization throughout the Evolutionary History of the Fig-Wasp Pollination Mutualism. Nat. Commun..

[7] Ware A.B., Compton S.G. (1992). Repeated Evolution of Elongate Multiporous Plate Sensilla in Female Fig Wasps (Hymenoptera: Agaonidae: Agaoninae). Proc. K. Ned. Akad. Wet..

[8] Benton R., Vannice K.S., Gomez-Diaz C., Vosshall L.B. (2009). Variant Ionotropic Glutamate Receptors as Chemosensory Receptors in Drosophila. Cell.

[9] Liu C., Liu Y., Walker W.B., Dong S., Wang G. (2013). Identification and Functional Characterization of Sex Pheromone Receptors in Beet Armyworm Spodoptera Exigua (Hübner). Insect Biochem. Mol. Biol..

[10] Ning C., Yang K., Xu M., Huang L.-Q., Wang C.-Z. (2016). Functional Validation of the Carbon Dioxide Receptor in Labial Palps of Helicoverpa Armigera Moths. Insect Biochem. Mol. Biol..

[11] Chang H., Liu Y., Ai D., Jiang X., Dong S., Wang G. (2017). A Pheromone Antagonist Regulates Optimal Mating Time in the Moth Helicoverpa Armigera. Curr. Biol..

[12] Abuin L., Bargeton B., Ulbrich M.H., Isacoff E.Y., Kellenberger S., Benton R. (2011). Functional Architecture of Olfactory Ionotropic Glutamate Receptors. Neuron.

[13] Ai M., Blais S., Park J.-Y., Min S., Neubert T.A., Suh G.S. (2013). Ionotropic Glutamate Receptors IR64a and IR8a Form a Functional Odorant Receptor Complex in Vivo in Drosophila. J. Neurosci..

[14] Rytz R., Croset V., Benton R. (2013). Ionotropic Receptors (IRs): Chemosensory Ionotropic Glutamate Receptors in Drosophila and Beyond. Insect Biochem. Mol. Biol..

[15] Koh T.-W., He Z., Gorur-Shandilya S., Menuz K., Larter N.K., Stewart S., Carlson J.R. (2014). The *Drosophila* IR20a Clade of Ionotropic Receptors Are Candidate Taste and Pheromone Receptors. Neuron.

[16] Ni L., Klein M., Svec K.V., Budelli G., Chang E.C., Ferrer A.J., Benton R., Samuel A.D., Garrity P.A. (2016). The Ionotropic Receptors IR21a and IR25a Mediate Cool Sensing in Drosophila. Elife.

[17] Ganguly A., Pang L., Duong V.-K., Lee A., Schoniger H., Varady E., Dahanukar A. (2017). A Molecular and Cellular Context-Dependent Role for Ir76b in Detection of Amino Acid Taste. Cell Rep..

[18] Xiao J.-H., Yue Z., Jia L.-Y., Yang X.-H., Niu L.-H., Wang Z., Zhang P., Sun B.-F., He S.-M., Li Z. (2013). Obligate Mutualism within a Host Drives the Extreme Specialization of a Fig Wasp Genome. Genome Biol..

[19] Chen L., Segar S.T., Chantarasuwan B., Wong D.-M., Wang R., Chen X., Yu H. (2021). Adaptation of Fig Wasps (Agaodinae) to Their Host Revealed by Large-Scale Transcriptomic Data. Insects.

[20] Zhang X., Wang G., Zhang S., Chen S., Wang Y., Wen P., Ma X., Shi Y., Qi R., Yang Y. (2020). Genomes of the Banyan Tree and Pollinator Wasp Provide Insights into Fig-Wasp Coevolution. Cell.

[21] Chen L., Feng C., Wang R., Nong X., Deng X., Chen X., Yu H. (2022). A Chromosome-Level Genome Assembly of the Pollinating Fig Wasp Valisia Javana. DNA Res..

[22] Croset V., Rytz R., Cummins S.F., Budd A., Brawand D., Kaessmann H., Gibson T.J., Benton R. (2010). Ancient Protostome Origin of Chemosensory Ionotropic Glutamate Receptors and the Evolution of Insect Taste and Olfaction. PLoS Genet..

[23] Mayer M.L., Ghosal A., Dolman N.P., Jane D.E. (2006). Crystal Structures of the Kainate Receptor GluR5 Ligand Binding Core Dimer with Novel GluR5-Selective Antagonists. J. Neurosci..

[24] Croset V., Schleyer M., Arguello J.R., Gerber B., Benton R. (2016). A Molecular and Neuronal Basis for Amino Acid Sensing in the Drosophila Larva. Sci. Rep..

[25] Hussain A., Zhang M., Üçpunar H.K., Svensson T., Quillery E., Gompel N., Ignell R., Grunwald Kadow I.C. (2016). Ionotropic Chemosensory Receptors Mediate the Taste and Smell of Polyamines. PLoS Biol..

[26] Silbering A.F., Rytz R., Grosjean Y., Abuin L., Ramdya P., Jefferis G.S., Benton R. (2011). Complementary Function and Integrated Wiring of the Evolutionarily Distinct Drosophila Olfactory Subsystems. J. Neurosci..

[27] Knecht Z.A., Silbering A.F., Ni L., Klein M., Budelli G., Bell R., Abuin L., Ferrer A.J., Samuel A.D., Benton R. (2016). Distinct Combinations of Variant Ionotropic Glutamate Receptors Mediate Thermosensation and Hygrosensation in *Drosophila*. Elife.

[28] Guo M., Krieger J., Große-Wilde E., Mißbach C., Zhang L., Breer H. (2013). Variant Ionotropic Receptors Are Expressed in Olfactory Sensory Neurons of Coeloconic Sensilla on the Antenna of the Desert Locust (*Schistocerca gregaria*). Int. J. Biol. Sci..

[29] Pitts R.J., Derryberry S.L., Zhang Z., Zwiebel L.J. (2017). Variant Ionotropic Receptors in the Malaria Vector Mosquito Anopheles Gambiae Tuned to Amines and Carboxylic Acids. Sci. Rep..

[30] Tang R., Jiang N.-J., Ning C., Li G.-C., Huang L.-Q., Wang C.-Z. (2020). The Olfactory Reception of Acetic Acid and Ionotropic Receptors in the Oriental Armyworm, *Mythimna Separata* Walker. Insect Biochem. Mol. Biol..

[31] De Bruyne M., Foster K., Carlson J.R. (2001). Odor Coding in the Drosophila Antenna. Neuron.

[32] Hallem E.A., Carlson J.R. (2006). Coding of Odors by a Receptor Repertoire. Cell.

[33] Yu H., Nong X., Fan S., Bhanumas C., Deng X., Wang R., Chen X., Compton S.G. (2023). Olfactory and Gustatory Receptor Genes in Fig Wasps: Evolutionary Insights from Comparative Studies. Gene.

[34] Quevillon E., Silventoinen V., Pillai S., Harte N., Mulder N., Apweiler R., Lopez R. (2005). InterProScan: Protein Domains Identifier. Nucleic Acids Res..

[35] Larkin M.A., Blackshields G., Brown N.P., Chenna R., McGettigan P.A., McWilliam H., Valentin F., Wallace I.M., Wilm A., Lopez R. (2007). Clustal W and Clustal X Version 2.0. Bioinformatics.

[36] Li W., Schuler M.A., Berenbaum M.R. (2003). Diversification of Furanocoumarin-Metabolizing Cytochrome P450 Monooxygenases in Two Papilionids: Specificity and Substrate Encounter Rate. Proc. Natl. Acad. Sci. USA.

[37] Kumar S., Stecher G., Tamura K. (2016). MEGA7: Molecular Evolutionary Genetics Analysis Version 7.0 for Bigger Datasets. Mol. Biol. Evol..

[38] Yang Z. (2007). PAML 4: Phylogenetic Analysis by Maximum Likelihood. Mol. Biol. Evol..

[39] Su Z.-H., Sasaki A., Kusumi J., Chou P.-A., Tzeng H.-Y., Li H.-Q., Yu H. (2022). Pollinator Sharing, Copollination, and Speciation by Host Shifting among Six Closely Related Dioecious Fig Species. Commun. Biol..

[40] Yu H., Tian E., Zheng L., Deng X., Cheng Y., Chen L., Wu W., Tanming W., Zhang D., Compton S.G. (2019). Multiple Parapatric Pollinators Have Radiated across a Continental Fig Tree Displaying Clinal Genetic Variation. Mol. Ecol..

[41] Wang R., Yang Y., Jing Y., Segar S.T., Zhang Y., Wang G., Chen J., Liu Q.-F., Chen S., Chen Y. (2021). Molecular Mechanisms of Mutualistic and Antagonistic Interactions in a Plant–Pollinator Association. Nat. Ecol. Evol..

[42] Karpe S.D., Dhingra S., Brockmann A., Sowdhamini R. (2017). Computational Genome-Wide Survey of Odorant Receptors from Two Solitary Bees *Dufourea Novaeangliae* (Hymenoptera: Halictidae) and *Habropoda Laboriosa* (Hymenoptera: Apidae). Sci. Rep..

[43] Cruaud A., Rønsted N., Chantarasuwan B., Chou L.S., Clement W.L., Couloux A., Cousins B., Genson G., Harrison R.D., Hanson P.E. (2012). An Extreme Case of Plant–Insect Codiversification: Figs and Fig-Pollinating Wasps. Syst. Biol..

[44] Yu H., Liao Y., Cheng Y., Jia Y., Compton S.G. (2021). More Examples of Breakdown the 1: 1 Partner Specificity between Figs and Fig Wasps. Bot. Stud..

[45] Getahun M.N., Wicher D., Hansson B.S., Olsson S.B. (2012). Temporal Response Dynamics of Drosophila Olfactory Sensory Neurons Depends on Receptor Type and Response Polarity. Front. Cell. Neurosci..

[46] Yao C.A., Ignell R., Carlson J.R. (2005). Chemosensory Coding by Neurons in the Coeloconic Sensilla of the *Drosophila antenna*. J. Neurosci..

[47] Prieto-Godino L.L., Rytz R., Bargeton B., Abuin L., Arguello J.R., Peraro M.D., Benton R. (2016). Olfactory Receptor Pseudo-Pseudogenes. Nature.

[48] Ai M., Min S., Grosjean Y., Leblanc C., Bell R., Benton R., Suh G.S. (2010). Acid Sensing by the Drosophila Olfactory System. Nature.

[49] Zhang Y.V., Ni J., Montell C. (2013). The Molecular Basis for Attractive Salt-Taste Coding in Drosophila. Science.

[50] Knecht Z.A., Silbering A.F., Cruz J., Yang L., Croset V., Benton R., Garrity P.A. (2017). Ionotropic Receptor-Dependent Moist and Dry Cells Control Hygrosensation in *Drosophila*. Elife.

[51] Gupta K., Dhawan R., Kajla M., Misra T., Kumar S., Gupta L. (2017). The evolutionary divergence of STAT transcription factor in different *Anopheles* species. Gene.

[52] Kumar V., Garg S., Gupta L., Gupta K., Diagne C.T., Misse D., Pompon J., Kumar S., Saxena V. (2021). Delineating the role of *Aedes aegypti* ABC transporter gene family during mosquito development and arboviral infection via transcriptome analyses. Pathogens.

